# Promoting Participation of Older Black Adults in Alzheimer’s Disease Research

**DOI:** 10.1093/geroni/igaf122.1200

**Published:** 2025-12-31

**Authors:** Rebecca Lassell, Christopher Carey

## Abstract

Symposium will be here

